# Acute Myocardial Infarction in a Seven-Year-Old Child With Type 1 Diabetes: A Rare Case Report

**DOI:** 10.7759/cureus.62909

**Published:** 2024-06-22

**Authors:** Hanane Hajaj, Aziza Elouali, Ayad Ghanam, Maria Rkain, Abdeladim Babakhouya

**Affiliations:** 1 Faculty of Medicine and Pharmacy, Mother and Child Health Laboratory, Mohammed First University, Oujda, MAR; 2 Department of Pediatrics, Mohammed VI University Hospital, Oujda, MAR

**Keywords:** electrocardiogram, child, acute chest pain, type 1 diabetes, myocardial infarction

## Abstract

Myocardial infarction (MI) is extremely rare in children and can have different etiologies, including congenital heart defects and Kawasaki disease. Cardiovascular disease (CVD) is the primary cause of death in patients with type 1 diabetes (T1D). Effective management of risk factors like blood pressure, cholesterol, and blood sugar levels is essential for individuals with T1D to mitigate the risk of cardiovascular complications, including MI. We present the case of a seven-year-old child diagnosed with type 1 diabetes one month before this admission, without any other notable medical history, who was admitted to the pediatric emergency department due to chest pain. The symptoms had begun two hours prior to admission. Upon arrival, the patient reported severe and persistent retrosternal constrictive chest pain radiating to the left arm without other associated signs, with a strictly normal clinical examination. An electrocardiogram (ECG) revealed typical ST segment elevation in inferior leads (II, III, and aVF) with reciprocal changes in V1 to V4. Troponin level was elevated at 7254 ng/l. Echocardiography revealed mild dilation of the left coronary artery (4 mm) and the right coronary artery (3 mm), while other radiological and laboratory investigations showed no abnormalities. The patient responded well to treatment with acetylsalicylic acid, clopidogrel, and heparin, resulting in a favorable outcome.

## Introduction

Type 1 diabetes mellitus (T1DM) stands as one of the most prevalent lifelong conditions affecting children [1]. Individuals with T1DM face an increased susceptibility to coronary heart disease (CHD), which encompasses acute myocardial infarction (AMI) [2-3]. Although MI is common in adults, it is extremely rare in children. The estimated incidence of AMI in adolescents was reported to be 157 cases per year or 6.6 events per one million patient-years [ 4]. The mortality rate from AMI is 0.2 deaths per 100,000 individuals aged 15 to 24 and less than 0.2 deaths per 100,000 in infants younger than one year [5]. It is a clinical condition that arises due to a sudden reduction or interruption in blood flow through the coronary vessels supplying the heart, which can occur for various reasons [6]. The most critical risk factors in neonates and infants include congenital heart disease, coronary artery abnormalities, and perinatal asphyxia [7-8]. Adolescents with T1DM also exhibit reduced functional exercise capacity and cardiovascular dysfunction [9]. We report a rare case of MI in a seven-year-old child with T1DM.

## Case presentation

A seven-year-old boy presented to the pediatric emergency department with chest pain that had begun two hours earlier. Upon his admission, he described severe and prolonged retrosternal constrictive chest pain radiating to the left arm without associated shortness of breath or syncope. There were no triggers, such as exertion or emotional stress. The patient had been diagnosed with T1DM one month prior to admission and was on a basal-bolus insulin regimen. There was no family history of T1DM or hypercholesterolemia, as well as no metabolic syndrome, cardiovascular disease (CVD), thrombophilia, or premature coronary artery disease. At the time of presentation, the patient was in a stable hemodynamic condition, with a blood pressure of 100/60 mmHg, a heart rate of 81 beats/min, a respiratory rate of 18 breaths/min, a body temperature of 36.9 °C, and a pulse oximetry reading of 98% on room air. His height was 133 cm (+1 SD), and his weight was 19 kg (-2 SD) at admission. Capillary blood glucose was 2.1 g/L (normal range: ≤2 g/L), and urinalysis revealed glucosuria (2+) without proteinuria or ketones. Physical examination findings were normal, with no evidence of congestive heart failure. Pulmonary auscultation was unremarkable, and cardiac auscultation revealed a regular pulse without any heart murmurs. Blood tests revealed no evidence of a biological inflammatory syndrome, with a leukocyte count of 18 G/L and a C-reactive protein level of 4.66 mg/L. The B-type natriuretic peptide (BNP) level was measured at 215 pg/mL (normal range: <100 pg/mL). Lipid profile results were as follows: total cholesterol 1.6 g/l, LDL cholesterol 1.07 g/l, HDL cholesterol 0.41 g/l, and triglycerides 0.59 g/l. The serum troponin level reached 7,254 ng/L within one hour of admission (Table 1). 

**Table 1:** Laboratory findings of our patient. LDL: low-density lipoprotein, HDL: high-density lipoprotein

Laboratory parameter	Test results	Reference range
Hemoglobin (g/dL)	12.8	11-13.5
White blood cell (/µL)	18120	4,000-10,000
Lymphocyte (/µL)	2870	2,000-4,000
Neutrophil (/µL)	14450	1,500-7,000
Platelets (/µL)	356000	150,000-400,000
C-reactive protein (mg/L)	4.66	<5
B-type natriuretic peptide ( pg/mL)	215	<100
Troponine (ng/L)	7254	0-40
Total cholesterol (g/L)	1.6	<1.7
LDL cholesterol (g/L)	1.07	<1.5
HDL cholesterol (g/L)	0.41	>0.4
Calcium (mg/L)	91	88-108
Potassium (mEq/L)	3.6	3.4-4.7
Sodium (mEq/L)	138	135-145

**Figure 1:**
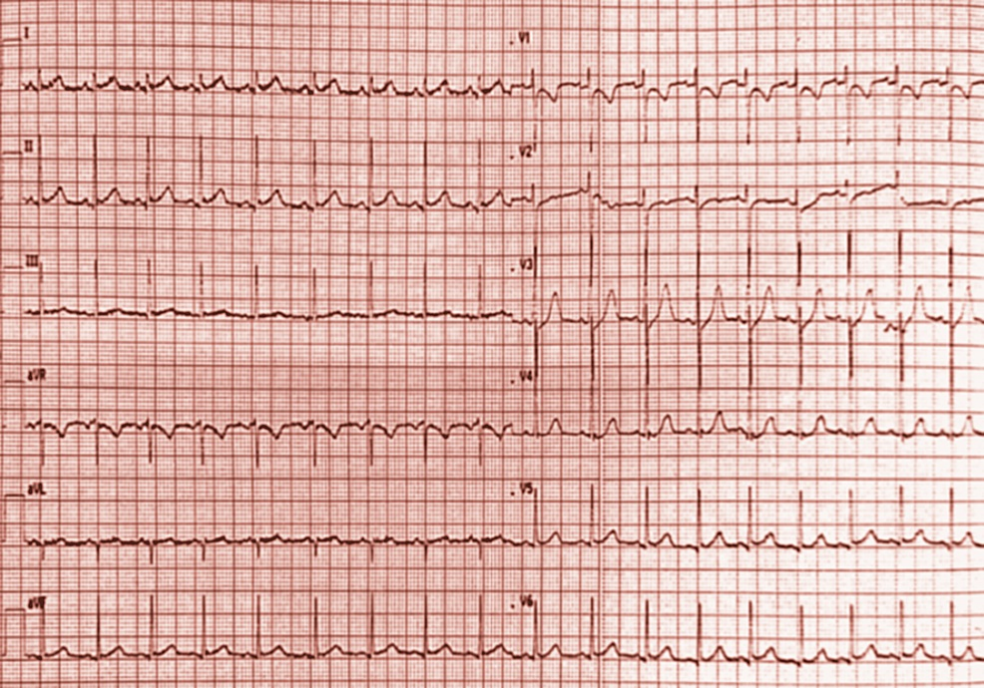
Electrocardiogram showed an ST segment elevation in inferior leads (II, III, and aVF) with reciprocal changes in V1 to V4.

The electrocardiogram performed within one hour of the patient's admission to the emergency room showed typical ST segment elevation in the inferior leads (II, III, and aVF) with reciprocal changes noted in V1 to V4 (Figure 1).

**Figure 2:**
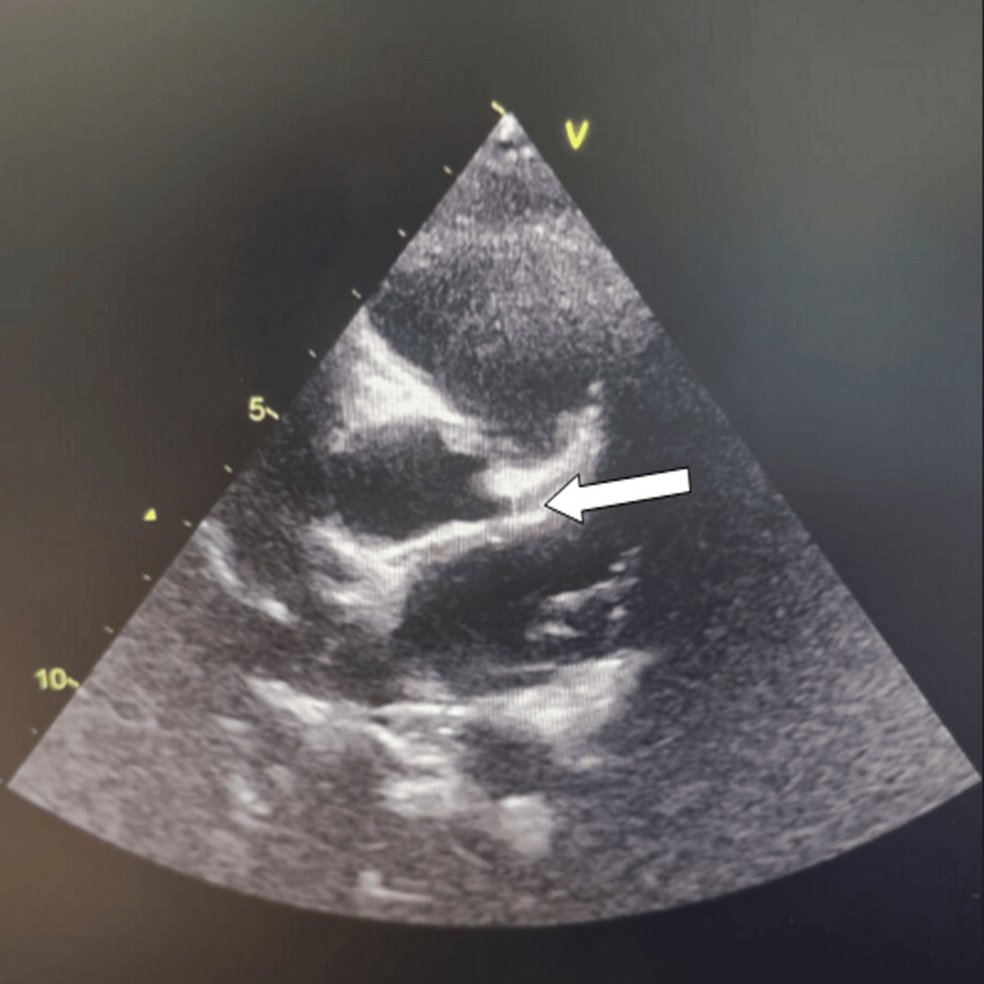
Echocardiography showed a dilated left coronary artery at 4mm.

Echocardiography revealed mild dilation of the left coronary artery measuring 4 mm and the right coronary artery measuring 3 mm (Figure 2).

Cardiac computed tomography was normal. Unfortunately, coronary angiography was not available. The patient received treatment with acetylsalicylic acid at 3 mg/kg/day, clopidogrel at 1 mg/Kg/day, and enoxaparin sodium at 0.2 IU/ 12 hours. In addition, the insulin regimen was adapted, and therapeutic education was provided with good evolution and normalization of ST-segment elevation by the second-day post-treatment, along with normalization of troponin levels within five days. On the eighth day, the patient was discharged with a stable hemodynamic condition.

## Discussion

MI is rare in children. According to reports, around 157 cases of AMI occur annually among adolescents [4]. In neonates, MI is mainly caused by perinatal asphyxia, while in infants, children, and adolescents, it frequently results from congenital abnormalities of coronary arteries. Other possible causes involve coronary arteritis (typically associated with Kawasaki disease), certain metabolic disorders (such as mucopolysaccharidosis, Fabry disease, gangliosidosis, and homocystinuria), myocardial bridging, coronary artery trauma due to thoracic injuries, mediastinal irradiation, chronic rejection post-heart transplantation, compression of coronary arteries by metastatic tumors, primary thrombocytosis, sickle cell disease, cocaine abuse, and surgical interventions for congenital heart diseases that require coronary artery reimplantation. In addition, coronary embolism can lead to MI in children [7].

Acute cardiac decompensation due to early atherosclerosis is more frequent in adults with T1DM compared to children with T1DM [10]. Finding the underlying cause can be challenging in patients without coronary abnormalities. Rawshani et al. demonstrated that individuals with T1DM who developed the disease before the age of 10 had a 30-fold increased risk of coronary heart disease and AMI in early adulthood [11]. While several studies suggest that diabetic ketoacidosis can lead to myocardial damage through multifactorial mechanisms [12], it is important to note that our patient did not present with diabetic ketoacidosis. The exact etiology of the MI in our patient remains unclear.

Batra et al. presented a case of MI in a child with diabetic ketoacidosis (DKA) [13]. They proposed that the accumulation of solute associated with hyperosmolarity alters blood flow and impairs erythrocyte flexibility, resulting in cardiovascular disturbances [13]. Rodriguez et al. conducted a comparison of CVD risk factor prevalence between children with T1DM and type 2 diabetes (T2D). They defined cardiovascular risk factors as HDL-C levels below 40 mg/dL, a waist circumference above the 90th percentile for age and sex, blood pressure above the 90th percentile for age, sex, and height (or the use of hypertension medication), and triglyceride levels above 110 mg/dL. Their findings revealed that 21% of diabetic children had at least two cardiovascular risk factors, with 92% of those with T2D and 14% of those with T1D being affected [14].

Studies have found a correlation between improved glycemic control and reduced CVD [15] and atherosclerosis in T1DM [16]. Hyperglycemia induces protein glycation, leading to the formation of advanced glycation end products, which play a role in the development of atherosclerosis [17-18].

Guidelines indicate that the management of children with AMI must be the same as for adults, despite differences in the underlying causes [19]. Therefore, pediatricians should remain vigilant and cautious regarding the presence of chest pain in a diabetic child, especially if it is accompanied by characteristic ST changes.

## Conclusions

In a diabetic child, chest pain should raise concerns for an MI, even among younger children who typically are not known to have coronary disease risk factors. MI in children may arise from various factors, necessitating distinct management strategies. Early identification and precise decisions are crucial factors in preventing morbidity and mortality.
